# Catarrhal Fever*Read before the East Texas Medico-Chirurgical Society at Palestine, November 29, 1900.

**Published:** 1901-03

**Authors:** J. M. Colley

**Affiliations:** Palestine, Texas


For Texas Medical Journal.

### Catarrhal Fever.*

BY J. M. COLLEY, M. D., PALESTINE, TEXAS.

This disease manifests itself by an imflammatory process which involves more or less of the mucous and sub-mucous tissues, also the white fibrous tissues of the human body.

---

*Read before the East Texas Medico-Chirurgical Society at Palestine, November 29, 1900.

This disease is found to vary very widely from a very simple form of fever lasting only a few days, possibly a day to a week or ten days and sometimes several weeks and may simulate typhoid fever.

The simple form, where the fever is slight, may or may not be ushered in by a chill. The aching and pain in the limbs is very slight, rise of temperature only a few degrees above normal, headache slight in the mild form of this disease.

Then again, we find a case which has had several days of malaise, afterwards to be followed by a severe chill when the temperature will rapidly rise high to 104° or 105° F. with intense headache, dry skin, pain very marked in limbs, loins and muscles generally, loss of appetite, bowels may be loose or constipated.

In the aggravated forms of this disease we may meet with many different complications, such, for example, as gastric or gastro-enteric form, then also bronchial or broncho-pneumonic involvements.

We sometimes find it not a little difficult in making a true differential diagnosis in every case we are called upon to treat.

We are told by some after trying quinine and the fever does not yield, that we have typhoid fever to contend with. My experience is that quinine is of very little benefit, if any, and am of the opinion that it will do no harm in some cases and is useful in aiding in making a diagnosis.

When we have high temperature during the night and remission during the day, together with sweating, we may safely discard typhoid fever.

In the treatment of this disease for the first few days I put my patients on the salicylates and the bromides, not to be continued longer than three or four days. If the disease does not yield, then give them chloride of iron, muriate of ammonia, strychnia, alcoholic stimulants, etc. I must say here that I give very little mercury, if any, in these cases, but rely mostly on saline cathartics, citrate of magnesia, phosphate of soda, etc.; treat symptoms as they develop.

